# Convection-Enhanced Delivery for Targeted Delivery of Antiglioma Agents: The Translational Experience

**DOI:** 10.1155/2013/107573

**Published:** 2013-02-14

**Authors:** Jonathan Yun, Robert J. Rothrock, Peter Canoll, Jeffrey N. Bruce

**Affiliations:** ^1^Gabriele Bartoli Brain Tumor Laboratory, Departments of Neurosurgery, Columbia University Medical Center, 1130 St. Nicholas Avenue Room 1001, New York, NY 10032, USA; ^2^Pathology and Cell Biology, Columbia University Medical Center, 1130 St. Nicholas Avenue Room 1001, New York, NY 10032, USA; ^3^Department of Neurological Surgery, Neurological Institute of New York, 710 West 168th Street, New York, NY 10032, USA

## Abstract

Recent improvements in the understanding of glioblastoma (GBM) have allowed for increased ability to develop specific, targeted therapies. In parallel, however, there is a need for effective methods of delivery to circumvent the therapeutic obstacles presented by the blood-brain barrier and systemic side effects. The ideal delivery system should allow for adequate targeting of the tumor while minimizing systemic exposure, applicability across a wide range of potential therapies, and have existing safe and efficacious systems that allow for widespread application. Though many alternatives to systemic delivery have been developed, this paper will focus on our experience with convection-enhanced delivery (CED) and our focus on translating this technology from pre-clinical studies to the treatment of human GBM.

## 1. Introduction

 Malignant gliomas are among the most pernicious of human tumors and are characterized as regionally invasive, usually recurring within two centimeters of their origin after resection [1]. Although many advances in treatment have been made, they have yielded only modest survival benefits [2]. Numerous chemotherapeutic drugs have demonstrated significant antitumor activity in preclinical studies, but often this effectiveness is not translated into clinical trials in humans. A major factor contributing to this is the limitation of systemic delivery, namely, the impermeability of the blood-brain barrier as well as dose-limiting toxicities of many compounds. This highlights the need for efficient, specific methods of delivery in the treatment of human GBM.

 The ideal delivery method would be one that achieves adequate coverage of the tumor volume while minimizing any unwanted toxicities. Optimal delivery requires three important components: the ability to target the tumor while minimizing local and systemic effects, applicability over a wide range of therapies, and a safe, efficacious method of continuous delivery with noninvasive methods to monitor volumes of distribution (Vd) of agents. In this paper, we describe our experience with convection-enhanced delivery (CED) across these three domains and highlight the translational goals of this work. The ultimate goal is to safely bring such systems and therapies to human trials, and eventually, to optimize these methods in clinical practice and establish standards of care.

## 2. Convection-Enhanced Delivery

Convection-enhanced delivery, pioneered by Bobo et al., delivers agents directly into the tumor and the surrounding parenchyma with continuous, positive-pressure infusion [3]. While other methods of delivery exist, such as through intraarterial and intrathecal routes, these are often limited by the blood-brain and blood-CSF barrier as well as unwanted toxicities. Furthermore, compared to diffusion-based drug delivery (i.e., carmustine wafers), convective delivery allows for larger volumes of distribution, as it is not limited by diffusive spread by concentration gradients [4]. Importantly, CED allows direct access to the tumor bed, achieving high local concentrations of drug with minimal systemic absorption.

One of the first therapeutic agents given via CED for malignant gliomas in a clinical trial was diphtheria toxin conjugated to transferrin (TF-CRM107) [9]. This pioneering clinical trial highlighted the capability of CED to maximize therapeutic effect while limiting toxicity, as adverse events were limited. Several Phase I and II studies with other targeted cytotoxins followed in succeeding years, including IL-4, IL-13, transforming growth factor (TGF)-a conjugated to pseudomonas exotoxin, herpes simplex virus (HSV)-1-tk gene-containing liposomes, and 131I-labeled chimeric monoclonal antibody to histone H1 (Cotara) [10–14]. These trials demonstrated tumor specificity and adequate agent distribution with adverse effects similarly limited to target tissue damage and minimal to no systemic toxicity. These trials were limited, however, by the specificity of the delivered agents, which targeted only a subpopulation of tumor cells. Prior to our clinical trial, paclitaxel was the only conventional chemotherapeutic agent delivered via CED in a clinical trial [15]. This was mainly because paclitaxel does not cross the BBB, thus allowing the investigators to demonstrate that DW-MRI could be used to approximate the volume of distribution of CED. The trial resulted in a large incidence (40%) of chemical meningitis, a major drawback to the choice of paclitaxel [15, 16]. Though these studies highlighted initial challenges in the application of CED, they demonstrated the importance of careful and rational selection of agents for use in this method of delivery.

## 3. Early Experiences: CED of Topotecan 

 Our initial experience with CED of antitumor agents utilized the cytotoxic agent topotecan. Topotecan is a camptothecin-class drug and acts as a topoisomerase-I inhibitor. It causes single-strand DNA breaks during DNA replication [17, 18]. This drug was selected after we demonstrated in vitro cytotoxicity against various malignant glioma cell lines [19]. Due to its activity in cells in the S-phase of division, topotecan is ideal for the treatment of mitotically active glioma cells in the setting of relatively quiescent brain tissue. Previous experience with topotecan demonstrated poor penetration of the blood-brain barrier and significant dose-limiting toxicities, limiting systemic administration [20–23]. However, these same properties make it an ideal drug for administration via CED.

 In addition, an important aspect of the choice of topotecan was its effect on a vital cellular process, namely, the role of topoisomerase I on DNA processes. This focus on conventional chemotherapeutic agents as opposed to targeted therapies allows for greater coverage of heterogeneous glioma subpopulations. While targeted therapies can be successful in eliminating a specific subpopulation of glioma cells that express a certain antigen, this provides a selective advantage for remaining neoplastic cells.

**Figure 1:**
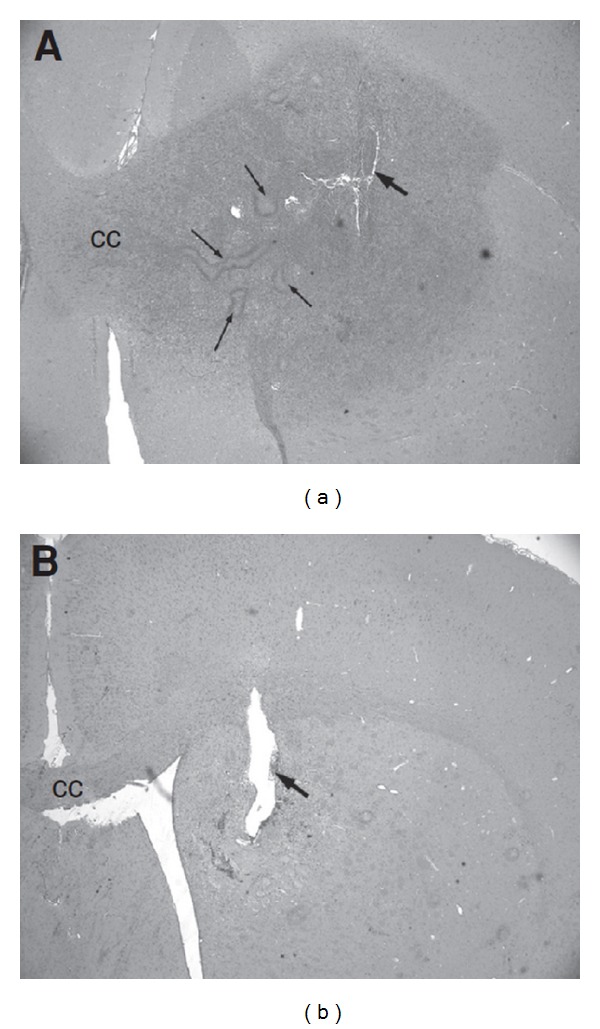
(a) The local delivery of PBS for 7 days into PDGF-expression retrovirus-induced tumor (large arrow—injection site) demonstrates a large proliferative lesion with notable pseudopalisading necrosis (small arrows) and invasion across the corpus callosum (CC). (b) The delivery of topotecan for 7 days results in significant decrease in tumor cells. (Figure reprinted with permission from Lopez et al. [5].)

Preclinical testing of topotecan that was performed in a model of glioblastoma was developed using a PDGF-B expressing retrovirus injected stereotactically into the adult white matter of rats to infect glial progenitors [19]. This resulted in the consistent development of tumors that closely resembled glioblastoma, with pseudopalisading necrosis, invasion, glomeruloid vascular proliferation, and survival of 14–19 days [24]. Topotecan was delivered using an implantable osmotic pump connected to an intracerebral infusion cannula (Alzet; Cupertino, CA) that was implanted into the tumor. A significant survival advantage was demonstrated in glioma-bearing rats treated with topotecan at concentrations significantly less than those used in systemic studies. Further, we found that animals treated for a longer period of time demonstrated increased survival benefit (1 d versus 4 d versus 7 d) [5] (Figure 1). Importantly, no adverse effects of the medication were observed. 

**Figure 2:**
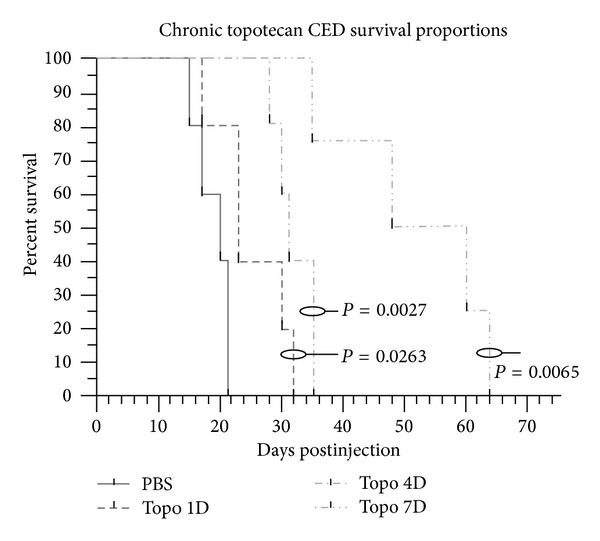
The local delivery of topotecan by convection-enhanced delivery resulted in significant survival advantage when compared to PBS treated controls. This effect was greater with longer periods of therapy. (Figure reprinted with permission from Lopez et al. [5].)

 Given the promising results of our preclinical studies, a Phase I, dose escalation clinical trial was undertaken to treat patients with recurrent glioblastoma with CED of topotecan. Topotecan was delivered to 18 patients with radiographically and pathologically confirmed recurrent high-grade glioma. While not primarily designed to test treatment efficacy, this clinical trial demonstrated that the CED of topotecan resulted in radiographic tumor regression in 69% of patients, with 25% demonstrating an early response, at a drug concentration nontoxic to normal brain with minimal drug-associated systemic toxicity [6] (Figure 2). This demonstrated that CED is an effective method of bypassing the blood-brain barrier to achieve targeted antitumor effect with minimal dose-limiting toxicities. Furthermore, topotecan proved to be a potent antitumor drug when delivered appropriately and directly to the tumor.

## 4. CED as a Platform to Assess Novel Antitumor Agents

**Figure 3:**
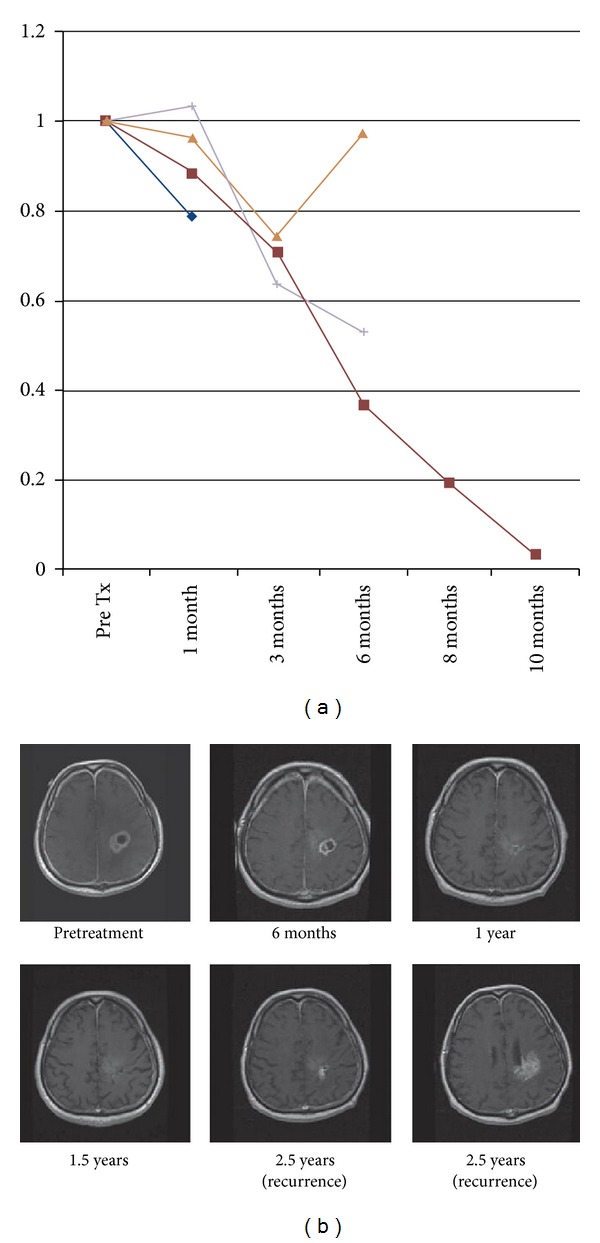
(a) 4 patients of the 16 treated demonstrated an immediate decrease in contrast enhancing volume following CED of topotecan, classified as early responders. (b) Serial T1 weighted, contrast MRI sequences from a selected patient demonstrating significant response with complete resolution at 1.5 years. Recurrence was noted at 2.5 years posttreatment. (Figure reprinted with permission from Bruce et al. [6].)

Various classes of drugs have been proposed as potential antitumor agents. CED is a valuable platform to assess the feasibility of administering these agents in vivo. For example, virus-mediated gene therapy has proven to be a promising modality to allow for tumor-specific delivery of gene constructs. However, the initial experience with these agents has been hindered by poor distribution [25]. We have found that CED is a viable method of distributing adenoviral particles widely across white matter tracts in a rodent model (Figure 3(a)). Furthermore, with the modification of these particles with supraparamagnetic iron oxide particles (Figure 5), we were able to characterize MRI signatures that would allow of the real-time monitoring of vector distribution (Figure 3(b)) [7].

CED allows for direct assessment of newly selected drugs by maximizing the specific delivery to the tumor, especially as the molecular understanding of human GBM continues to identify new potential targets. Based on the work of Verhaak et al., GBM has been subdivided based on 4 distinct molecular signatures: classical, neural, proneural, and mesenchymal [26]. Our group has developed a mouse model of glioma which is induced by injecting a retrovirus that expresses PDGF-B and cre recombinase into the subcortical white matter of transgenic mice that harbor floxed alleles of the tumor suppressor genes, PTEN and p53. We found that the expression profile of these tumors closely resembles the proneural subtype of GBM [27]. This model provides a powerful tool to assess therapies in treating this specific subtype of GBM. Also, by understanding the molecular profile of this subtype, rational selection of antitumor agents can be pursued.

Within human TCGA data, we found that topoisomerases are differentially expressed across the 4 GBM subtypes, with proneural subtype showing the highest levels of both TOP2a and TOP2b expressions. The elevated expression of topoisomerase II seen in the proneural subgroup suggested that these tumors might be particularly sensitive to inhibitors of topoisomerase II. We also found elevated expression of topoisomerase II compared to topoisomerase I in our murine model of proneural GBM ( Carminucci et al. [28]).

Based on these findings, we hypothesized that etoposide, a topoisomerase II inhibitor, would exhibit effective cytotoxicity against the proneural subtype of GBM. We are currently undergoing preclinical testing with local, continuous delivery of etoposide in our mouse model of proneural GBM, which demonstrates significant antitumor activity and prolonged survival (Carminucci et al. [28]). We hope to translate these findings into early Phase I and II trials and to assess clinical and radiographic response with an understanding of the specific molecular subtypes of tumors treated.

## 5. Prolonged CED with Implantable Subcutaneous Pumps

 In our initial clinical trial, we demonstrated the ability of CED to deliver and effectively treat tumors with chemotherapy, all while bypassing the blood brain barrier and minimizing systemic toxicity. These clinical studies utilized externalized catheters, which, due to an increasing risk of infection with longer placement, shortened the treatment period to 4 days [6]. As mentioned above, in our rodent model, we demonstrated that prolonged delivery of topotecan is associated with increased survival. Therefore, we sought to develop a system for prolonged delivery that could be safely applied in the clinical setting.

To this end, we have employed an implantable subcutaneous pump (Synchromed II, Medtronic; Minneapolis, MN), already FDA approved for the treatment of spasticity and chronic pain. To assess this system in the pre-clinical setting, the pig model was used due to the larger size of the brain in comparison to the rodent model and its similarity to human gray/white matter composition. The pump was implanted into a subcutaneous pocket in the pig's back, and silastic catheter was tunneled subcutaneously and inserted into the frontal white matter. The reservoir was filled with a mixture of topotecan and/or gadolinium and was infused over a period of 10 days. The volumes of distribution were followed with serial MRI, and safety and toxicity were assessed on a daily basis [8]. 

**Figure 4:**
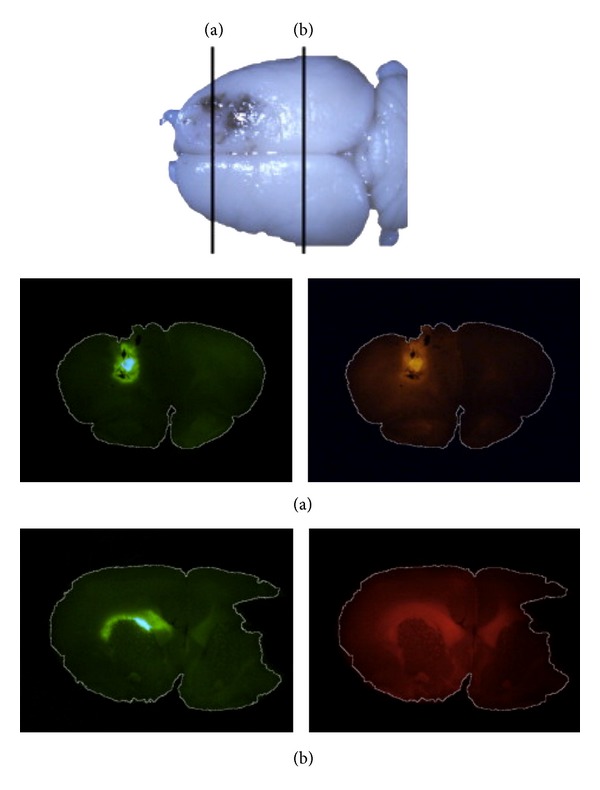
Infusion of an adenoviral vector (Ad5) expressing GFP and rhodamine-dextran demonstrates distribution of the vector throughout the ipsilateral white matter at (a) rostral and (b) caudal sections of the brain. (Figure reprinted with permission from Yun et al. [7].)

**Figure 5:**
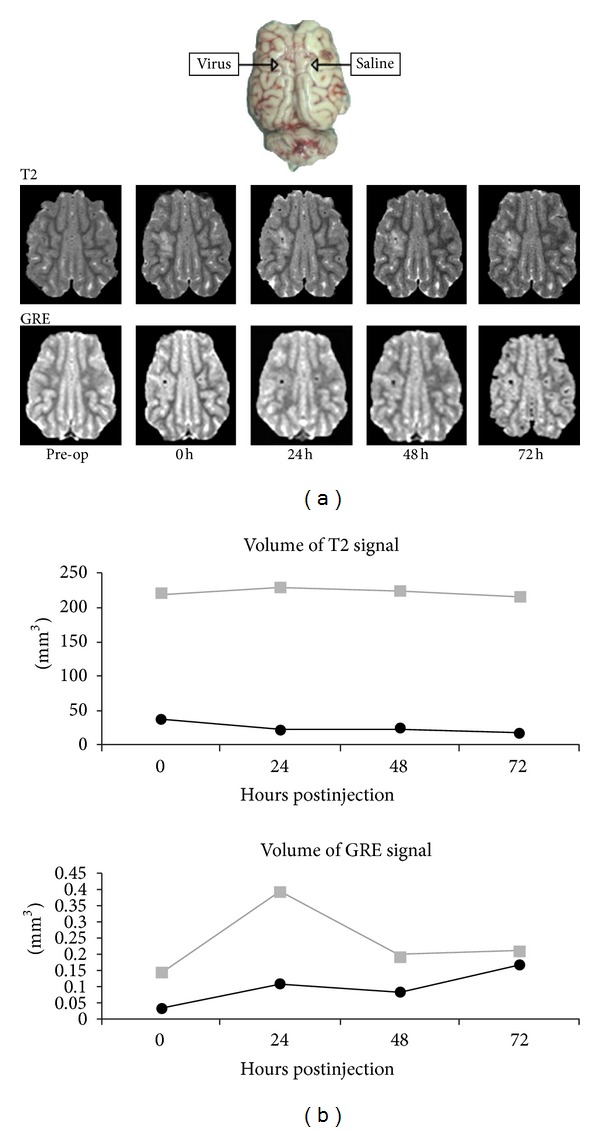
(a) Local injection of a superparamagnetic iron oxide nanoparticle labeled adenovirus demonstrates a T2-intense, GRE positive signal at the site of injection when compared to the contralateral, saline-injected side. (b) Volume analysis demonstrates a greater T2-intense volume with adenovirus injection when compared to saline. (Figure reprinted with permission from Yun et al. [7].)

 In this study, we demonstrated safety of topotecan with prolonged intracerebral infusion in nontumor bearing animals. Furthermore, topotecan retained its antitumor bioactivity after prolonged exposure to physiologic conditions. We demonstrated stability of the volume of distribution of gadolinium with prolonged delivery, with rapid reabsorption of contrast following cessation of infusion [8] (Figure 4). Along with the tolerability of the implanted pump, these findings provide justification for translation of this system to clinical trials, and we hope to employ this system for the treatment of human gliomas. 

## 6. Challenges 

**Figure 6:**
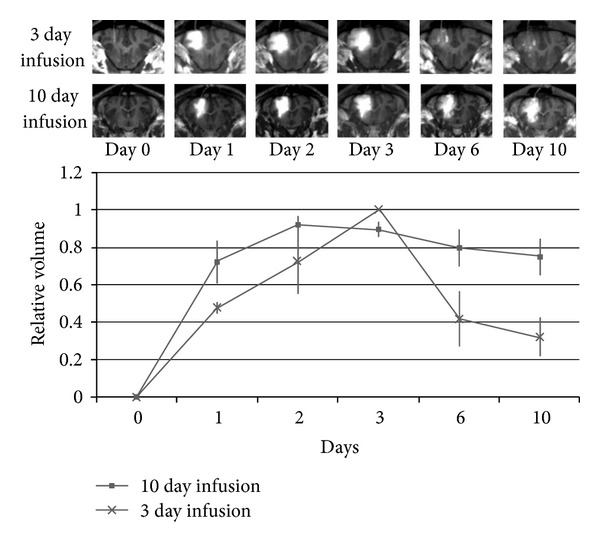
Prolonged infusion (10 d) with an implanted subcutaneous pump results in stable volumes of distribution. The maximum relative volume was reached 2-3 days after infusion was initiated. With infusion discontinued at day 3, enhancing volume is seen to dissipate. (Figure reprinted with permission from Sonabend et al. [8].)

 The administration of therapeutics via CED is not without its challenges, most notably the leakage of refluxed infusate along the catheter [29]. Other risks include infection, as well as those related to the drug, including potential systemic events if the agent is able to cross the blood-brain barrier. In our experience, however, the biologically active doses of the therapeutic agent administered via CED are well below systemic dose limiting toxicities. As Saito et al. have demonstrated, the volume of distribution (Vd) (Figure 6) achieved by CED is dependent on multiple compound specific factors (i.e., lipophilicity), as well as anatomical variables (i.e., tumor architecture and white matter tracks) [30]. The potential volumes achievable with CED, however, are greater than the volumes achieved by implantable wafers and diffusion-based therapies [31]. 

## 7. Discussion

Convection-enhanced delivery provides a method of local delivery of antitumor agents directly to the tumor and the surrounding infiltrative edges. Benefits of this system include volumes of distribution not limited by the physical characteristics of the drug or diffusive spread along concentration gradients [4]. This allows for the administration of a wide range of antitumor drugs that have been previously limited by systemic toxicities and poor distribution.

 Further, this method of delivery allows for greater flexibility with drug development and selection, as the effects of the blood brain barrier and systemic metabolism are minimized with direct, targeted delivery to the tumor. With the development of an implantable system that allows for prolonged delivery, it is conceivable that GBM can be treated chronically with single or multiple, sequential agents. Thus, our experience with CED demonstrates the ability to target tumors for the local delivery of a wide range of therapies, with systems that allow for a safe transition to the treatment of patients.
